# Comparison of standing postural control and gait parameters in people with and without chronic low back pain: a cross-sectional case–control study

**DOI:** 10.1136/bmjsem-2017-000286

**Published:** 2018-01-23

**Authors:** Catharine Siân MacRae, Duncan Critchley, Jeremy S Lewis, Adam Shortland

**Affiliations:** 1 College of Health and Life Sciences, Brunel University, Uxbridge, UK; 2 Therapy Services, Chelsea and Westminster Hospital NHS Foundation Trust, London, UK; 3 Division of Health and Social Care Research, Academic Department of Physiotherapy, King’s College London, London, UK; 4 Department of Allied Health Professions, University of Hertfordshire, Hatfield, UK; 5 Musculoskeletal Services, Central London Community Healthcare NHS Foundation Trust, London, UK; 6 One Small Step Gait Laboratory, Guy’s and St Thomas’ NHS Foundation Trust, London, UK; 7 Biomedical Engineering, King’s College London, London, UK

**Keywords:** lumbar spine, back injuries, exercise rehabilitation, gait analysis

## Abstract

**Objective:**

Differences in postural control and gait have been identified between people with and without chronic low back pain (CLBP); however, many previous studies present data from small samples, or have used methodologies with questionable reliability. This study, employing robust methodology, hypothesised that there would be a difference in postural control, and spatiotemporal parameters of gait in people with CLBP compared with asymptomatic individuals.

**Methods:**

This cross-sectional case–control study age-matched and gender-matched 16 CLBP and 16 asymptomatic participants. Participants were assessed barefoot (1) standing, over three 40 s trials, under four posture challenging conditions (2) during gait. Primary outcome was postural stability (assessed by root mean squared error of centre of pressure (CoP) displacement (CoP_RMSEAP_) and mean CoP velocity (CoP_VELAP_), both in the anteroposterior direction); gait outcomes were hip range of movement and peak moments, walking speed, cadence and stride length, assessed using force plates and a motion analysis system.

**Results:**

There were no differences between groups in CoP_RMSEAP_ (P=0.26), or CoP_VELAP_ (P=0.60) for any standing condition. During gait, no differences were observed between groups for spatiotemporal parameters, maximum, minimum and total ranges of hip movement, or peak hip flexor or extensor moments in the sagittal plane.

**Conclusions:**

In contrast to previous research, this study suggests that people with mild to moderate CLBP present with similar standing postural control, and parameters of gait to asymptomatic individuals. Treatments directed at influencing postural stability (eg, standing on a wobble board) or specific parameters of gait may be an unnecessary addition to a treatment programme.

**What are the new findings?**

- People with mild to moderate chronic low back pain (CLBP) presented with similar standing postural control to asymptomatic individuals.
- During gait, spatiotemporal parameters were similar in people with and without CLBP.
- During gait, hip kinetics and kinematics were similar in people with and without CLBP.
- Treatments directed at influencing postural stability or specific parameters of gait may be an unnecessary addition to a treatment programme for people with CLBP.

## Introduction

Differences in postural control^1–4^ and gait^5–10^ have been identified between people with and without chronic low back pain (CLBP). During more challenging standing conditions people with CLBP have demonstrated increased centre of pressure (CoP) displacements and velocities,^1–4^ indicative of poorer postural stability.^11 12^ A systematic review investigating difference in standing postural sway between those with and without CLBP reports inconsistent findings.^13^ Although the majority of studies reported an increased postural sway in people with LBP, evidence from fewer studies, many with larger sample sizes and more robust methodologies, demonstrated no difference between groups.^13^ Hence, whether a true difference exists remains unclear.

During gait, people with CLBP have demonstrated reduced self-selected walking speed,^5–8^ stride time,^9 10^ stride length^5 6^ and range of hip movement^9^ compared with people without back pain. Due to the proposed decrease in stride length, walking speed and hip range of movement, hip joint moments are also likely to be decreased in people with CLBP compared with people without.^14^ Researchers have proposed that such gait changes may be an attempt by the individual to reduce pain by reducing: ground reaction forces at heel strike^15^; excessive muscle activity; or joint movement.^16^ Alternatively, differences may be a result of altered proprioceptive feedback^17^ or psychological factors associated with CLBP, such as anxiety, fear avoidance and catastrophising.^18^ Psychological factors may lead to adaptation of normal physical activities, such as fast walking, due to the fear of increasing pain. Although gait alterations may initially be protective, such alterations may induce mechanical problems in the long term, for example, a slower walking produces longer periods of loading on the lumbar spine during gait,^19^ which may be detrimental to spinal structures in the long term, whereas shorter periods of loading, thought to be less detrimental, occur during faster walking.^19^

These differences in postural control^1–4^ and gait^5–10^ have been proposed as contributing factors to the presence and recurrent nature of CLBP.^1 4 15^ However, previous studies have used: small sample sizes^2 3^ (possibly introducing a type 2 error); methodological design likely to result in low reliability of data, for example, analysing data from one trial instead of multiple trials^1 5 8 9^; outcomes that have demonstrated poor reliability; or provide results not representative of the general population (eg, all or mainly male participants^8 9^; or walking on a treadmill as opposed to on normal ground^7 9 10^).

This study aimed to add to current research by using a more reliable and valid methodology to determine whether participants with CLBP have similar or different barefoot standing postural control, and gait parameters, when compared with age-matched and gender-matched asymptomatic participants. This methodology has previously been published as part of a randomsied trial.^20^ The following hypotheses were investigated:

H_1_: The CLBP group will demonstrate greater postural instability when compared with the asymptomatic group during more challenging standing conditions.

H_2_: Reduced self-selected walking speed, cadence and step length will be observed in people with CLBP compared with asymptomatic individuals.

H_3_: During gait, people with CLBP will present with reduced peak hip extensor moments during stance phase and reduced hip range of movement compared with asymptomatic individuals.

## Methods

This cross-sectional case–control study compared barefoot standing balance and gait data from CLBP participants with that from age-matched and gender-matched asymptomatic participants. This methodology has previously been published as part of a randomised trial.[new ref]

### Participant recruitment

A convenience sample of asymptomatic adults was recruited from acquaintances and colleagues of the investigators. Participants with CLBP were recruited from four Physiotherapy Departments in London (UK) (three National Health Service hospitals, one private physiotherapy practice) following clinical referral from general practitioners and consultants as part of a previously reported randomised controlled trial (RCT).^21^ During the second half of the recruitment period of the RCT, 55 participants were asked to participate in the current study, 38 of which showed interest. Eighteen participants could not attend the session in the main due to work commitments. Of the remaining 20 participants, only 16 could be matched by age and gender to our asymptomatic group. Inclusion criteria for symptomatic individuals were: aged 18–65 years, with a 3-month or greater history of LBP. Exclusion criteria were constant non-mechanical LBP, lumbar radiculopathy, known spondylolisthesis, spinal stenosis or inflammatory back pain, specific spinal diagnosis inappropriate for physiotherapy interventions (eg, spinal fracture or infection); any condition inappropriate for exercise physiotherapy (eg, severe cardiovascular or metabolic disease) or for wearing rocker sole footwear (eg, Morton’s neuroma, peripheral neuropathy). Potential asymptomatic participants were contacted via email including the Participant Information Sheet, and were asked to contact CSM if they wished to partake in the study. Asymptomatic participants reported no history of LBP in the last year, were required to meet all other inclusion and exclusion criteria presented above. As increasing age is a contributing factor to poorer postural stability^22^ and gender may influence postural control,^23^ hence potential confounding factors, asymptomatic participants were matched by age and gender to symptomatic participants. An age range of 2 years above or below the age of the ‘matched’ CLBP participant was classed as acceptable. Sixteen asymptomatic participants were consented into the study.

### Data collection

Data collection occurred at the ‘One Small Step Gait Laboratory’, Guys’ Hospital, London. Demographic and pain scores (numerical rating scale) representing their level of back pain on the day of assessment were recorded from all participants.

#### Biomechanical assessment

Participants were assessed wearing short trousers and vest or no top. Participants’ anthropometric measurements (pelvic width; leg length; knee width; ankle width; height; and weight) were recorded to inform the mechanical model formulated for each participant in Vicon’s Nexus (V.1.8.1) motion capture software (Vicon Motions Systems, Oxford, UK). The motion analysis system consisted of seven cameras, capturing retroreflective markers in three-dimensional space at a rate of 120 Hz.

**Figure 1:**
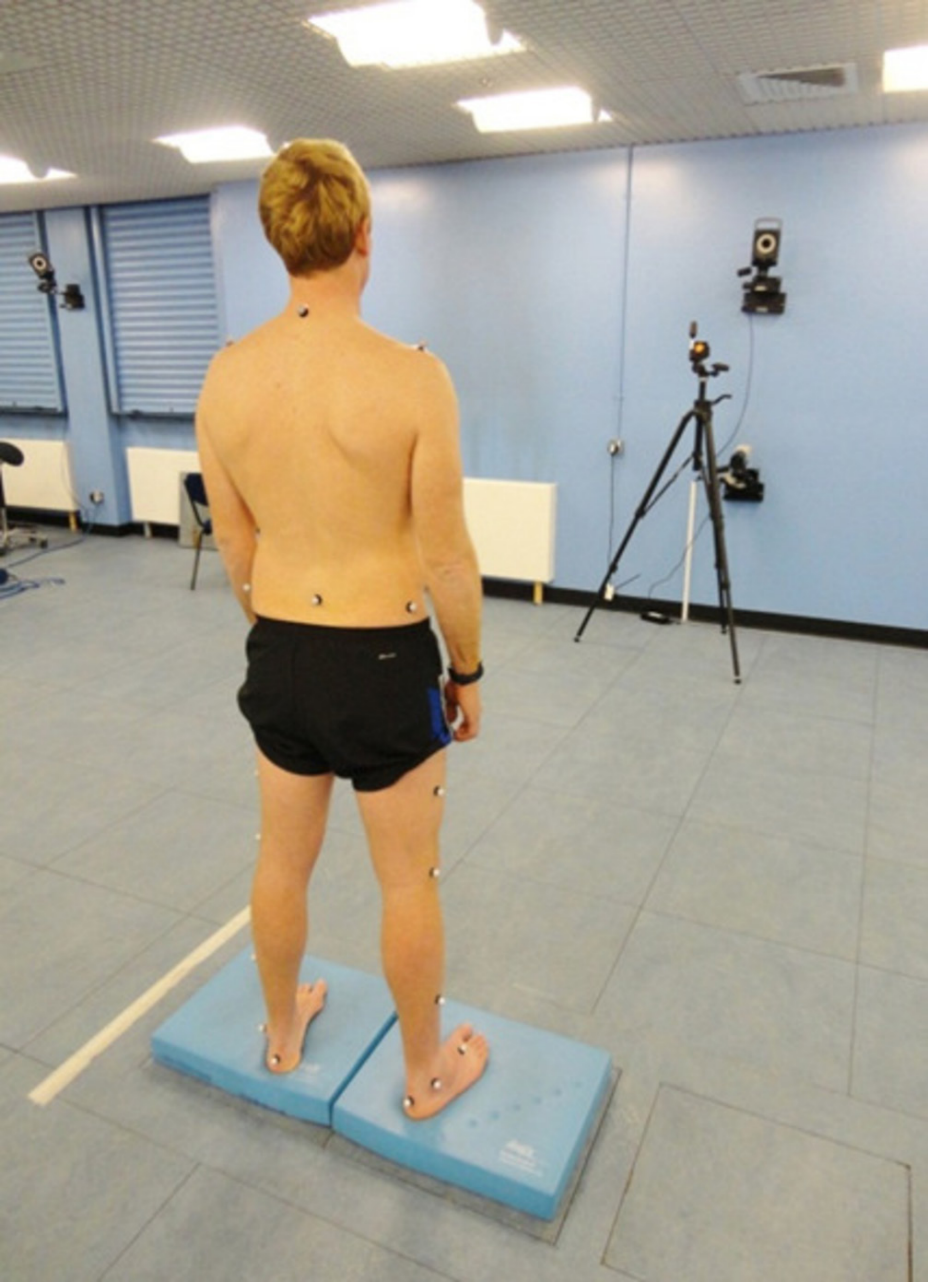
Participant with infrared reflective markers in situ standing on foam cushions overlying force plates.

Seventeen infrared reflective markers (14 mm diameter) were positioned on each participant by an experienced researcher (AS).^24–26^ The modified Helen Hayes marker set was implemented^27^ with additional markers on bilateral iliac crests, and posterior calcanei (figure 1).

#### Postural stability in standing

Participants were assessed barefoot, feet approximately pelvis width apart and on adjacent force plates (FP5000, AMTI, Massachusetts, USA), during four posture challenging standing conditions involving manipulation of visual input and support surface: (1) firm surface, eyes open; (2) firm surface, eyes closed; (3) compliant surface, eyes open; (4) compliant surface, eyes closed. Compliant surface was achieved by placing an AirexTM cushion (48.5×40.0×6.4 cm, 0.7 kg, density 38.6 kg/m^−3^, closed-cell foam) (l-group, St Louis, MO) over each force plate (figure 1).

Participants were instructed to keep their eyes focused on a red sticker at eye height on a tripod 3 m in front of them.^28^ Participants were assessed for three 40 s trials (shown to produce acceptable reliability^29^) for each standing condition. The middle 30 s of each trial was analysed to avoid possible initial sway errors, effects of participant fatigue or anticipation of a trial ending. Each participant received the same instructions at the start of each trial:

When I say ‘Go’ I want you to stand and maintain your balance until you hear the instruction to rest. Each trial will last for 40 s. Focus on the red sticker on the tripod ahead of you. Keep your arms relaxed by your sides.

A rest period of 20 s occurred between each trial. Sufficient trials were performed to provide three valid sets of data. A test was invalidated if the participant moved their foot position during the test, changed their arm starting position, or opened their eyes during an eyes-closed task.

#### Assessment of gait

Participants were asked to walk barefoot, at a pace that felt comfortable to them, from one end of the laboratory to the other, in a line which passed over three force plates. Each participant received the same instructions:

When I say go I want you to walk in a straight line to the marker at the other end of the room. Walk at a pace that feels comfortable to you.

Participants continued walking the length of the laboratory until CSM had observed three clear force plate strikes (heel strike and toe-off occurring with the foot making contact with one plate only, without contacting the plate with the contralateral foot) for each foot. The biomechanical assessment lasted approximately 30 min.

### Outcome measures

The following postural stability primary outcomes were assessed during standing: (1) root mean squared error and (2) velocity of the CoP in the anteroposterior direction (CoP_RMSEAP_ and CoP_VELAP_, respectively, online supplementary appendix 1). CoP is a term that refers to the mean position of the forces acting under the feet at any instant in time. The RMSE (or SD) of the CoP position reflects the spread of these measurements over a particular time interval (in this case 30 s). The CoP_VELAP_ refers to the mean displacement of the CoP in the anterior-posterior direction, divided by the sample time (1/1080 s) over the course of the 30 s trial. Reliability of CoP_VEL_ has been reported as excellent (intraclass correlation (ICC) 0.8–0.95) and CoP_RMSE_ reported as fair to good (ICC 0.32–0.58) for studies employing similar number of trials and trial durations as the current study.^12^

10.1136/bmjsem-2017-000286.supp1 Supplementary file 1

The following outcome measures were assessed during gait: self-selected walking speed, stride length, cadence, maximum, minimum and total hip range of movement, peak hip flexor and extensor moments.

### Data extraction

Force plate data (forces and moments) captured at 1080 Hz and filtered with a low pass Woltering filter (mean SE 10 mm^2^) were exported into Vicon’s Nexus software (V.1.8.1) to calculate biomechanical outcome measures.

Industry-standard motion capture files (.c3d) containing force data were extracted. Force plate data were filtered with a low pass (10 Hz) Butterworth filter. CoP parameters were calculated using a proprietary programme written in Visual Basic for Applications (Microsoft Excel, Reading, UK).

### Sample size

A sample size calculation was not conducted due to the lack of reported data of minimal clinically important difference (MCID) for the primary outcome measures (CoP parameters). This study aimed to recruit 20 asymptomatic participants age-matched and gender-matched to symptomatic participants recruited by the authors in a previous RCT.^21^

### Data analysis

Independent t-tests for parametric, or Mann-Whitney U tests for non-parametric data, were applied to determine differences between groups for demographic data and gait outcomes. A mixed-repeated measures analysis of variance with two within-subject factors each with two levels—vision (eyes open and eyes closed) and support surface (firm and compliant)—determined possible significant main effects and interactions of the two groups for CoP variables. The alpha level for determining statistical significance was set at 0.05. Data were analysed using IBM SPSS V.20.0.0 (IBM). Results are presented as means (SD) unless otherwise stated.

## Results

### Recruitment and retention

During the recruitment period (June 2010 to November 2011), 16 asymptomatic participants were age-matched and gender-matched with 16 CLBP participants. The recruitment of matched asymptomatic participants, over the age of 50 years, who had not experienced LBP over the past 12 months proved difficult. This prevented recruitment of the planned sample size of 20 participants per group. There was 100% retention with all 32 participants completing the data collection process.

### Baseline characteristics of participants

Demographic characteristics of CLBP and asymptomatic individuals are presented in table 1. No differences were observed between groups other than self-reported pain scores. Participants with CLBP reported mild to moderate pain with a numerical rating score range of 3–8, and a mean duration of symptoms of 6.17 (SD 7.59, range 0.25–31) years.

**Table 1:** Demographic data for chronic low back pain and asymptomatic participants

	Asymptomatic participants (n=16)	Low back pain participants (n=16)	P value
Gender			
Male	8 (50.0%)*	8 (50.0%)*	1.00†
Female	8 (50.0%)*	8 (50.0%)*	
Age (years)	37.3 (11.1)	36.8 (10.1)	0.90
Weight (kg)	76.3 (13.6)	73.4 (10.6)	0.52
Height (cm)	173.4 (9.3)	173.4 (8.9)	1.00
Numerical rating score for pain (0–10; 0=best)	0.0 (0.0)	5.9 (1.5)	0.00

Summary measures represent means (SD) or numbers (percentages).

*Numbers (percentages).

†Χ^2^ test, otherwise independent t-test.

### CoP parameters during standing

Table 2 presents data for the anteroposterior CoP parameter data for CLBP and asymptomatic participants during different standing conditions. There were no differences between the groups in CoP_RMSEAP_, or CoP_VELAP_ for any of the four standing conditions (F(2.35, 70.38)=1.39, P=0.26, η^2^=0.04; F(1.76, 52.87)=0.47, P=0.60, η^2^=0.02, respectively).

**Table 2:** Anteroposterior centre of pressure parameters for chronic low back pain and asymptomatic participants during different standing conditions

		CoP_RMSEAP_ (mm)	CoP_VELAP_ (mm/s)
Eyes open, firm surface	Asymptomatic	3.76 (0.84)	6.57 (1.09)
	Chronic low back pain	4.21 (1.88)	7.14 (1.52)
Eyes closed, firm surface	Asymptomatic	3.93 (1.47)	7.14 (1.10)
	Chronic low back pain	4.23 (1.38)	7.39 (1.24)
Eyes open, compliant surface	Asymptomatic	8.29 (1.70)	10.97 (1.78)
	Chronic low back pain	9.10 (2.95)	12.57 (3.96)
Eyes closed, complaint surface	Asymptomatic	8.93 (1.45)	17.15 (4.29)
	Chronic low back pain	10.56 (2.85)	17.98 (4.38)

Summary measures represent means (SD).

AP, anteroposterior; CoP, centre of pressure; RMSE, root mean squared error; VEL, velocity.

### Spatiotemporal parameters of gait

No differences were observed between groups for any of the spatiotemporal gait parameters assessed (table 3).

**Table 3:** Spatiotemporal parameters of gait in chronic low back pain and asymptomatic individuals

	Asymptomatic group	Chronic low back pain group	P value
Walking speed (m/s)	1.32 (0.13)	1.25 (0.20)	0.26
Cadence (steps per minute)	115.14 (6.59)	112.43 (11.81)	0.42
Stride length (m)	1.38 (0.12)	1.33 (0.13)	0.33

Summary measures represent means (SD). Analysis by independent t-test.

### Hip moments and range of movement during gait

No differences were detected between groups for maximum, minimum and total ranges of movement at the hip in the sagittal plane during gait (table 4). No differences were observed between groups for peak hip flexor or extensor moments during gait (table 4).

**Table 4:** Sagittal plane hip range of movement and peak hip joint moments during gait in people with chronic low back pain and asymptomatic individuals

	Asymptomatic	Chronic low back pain	P value
Left maximum hip flexion (°)	34.35 (5.55)	33.70 (8.55)	0.78
Right maximum hip flexion (°)	34.46 (4.51)	33.82 (9.17)	0.79
Left maximum hip extension (°)	−9.71 (7.39)	−10.44 (9.02)	0.80
Right maximum hip extension (°)	−9.40 (6.67)	−9.12 (8.74)	0.92
Left hip range of movement (°)	44.07 (3.94)	44.14 (4.79)	0.97
Left hip extensor moment (Nmm/kg)	1029.30 (329.38)	955.80 (429.78)	0.58
Right hip extensor moment (Nmm/kg)	960.99 (235.24)	1029.57 (460.62)	0.94*
Left hip flexor moment (Nmm/kg)	−990.76 (184.25)	−1098.07 (231.85)	0.14
Right hip flexor moment (Nmm/kg)	−1041.87 (174.80)	−977.77 (194.64)	0.31

Summary measures represent means (SD).

*Represents Mann-Whitney U test for non-parametric data, otherwise independent t-test conducted.

Nmm/kg, Newton-millimetre/kilogram.

## Discussion

In contrast to much other research, the current findings suggest that postural control during standing, and the kinetics, kinematics and spatiotemporal parameters of gait do not differ between people with CLBP of a mild to moderate intensity and asymptomatic individuals. There were no differences between people with and without CLBP in postural stability during all standing conditions assessed. During barefoot gait, both groups presented with similar peak hip moments and ranges of movement, and spatiotemporal parameters of gait. Hence, all stated hypotheses are rejected.

### CoP parameters

There was no difference in postural stability between CLBP and asymptomatic individuals during stable and more challenging standing conditions. These findings differ from previous research^1–4^ possibly due to methodological variation. della Volpe *et al* 2 assessed a smaller sample (n=12 per group) with an ‘instrumented platform system’, constructed of a moveable support surface and moveable visual surround likely to present participants with a greater postural challenge. This may contribute to the reduced postural stability observed in the CLBP group in their study.^2^ Brumagne *et al* 1 assessed a larger sample size than the current study (n=45); however, trials were only repeated once—the current study averaged three trials per standing condition, likely to increase reliability of data.^11^ Although Brumagne *et al* 1 reported reduced postural stability in the CLBP group during more challenging standing conditions, the between-group difference in CoP_RMSEAP_ was 1.8 mm, and the P value, 0.046–bordering on non-significance. In the current study, the non-significant difference in CoP_RMSEAP_ between the symptomatic and asymptomatic groups during the most challenging postural condition was 1.76 mm. Although Brumagne *et al* 1 demonstrated statistical significance, based on the very similar yet non-significant between-group difference in CoP displacement found in the current study (and in the absence of knowledge regarding cause or effect), it seems unlikely that such a minimal difference in CoP_RMSEAP_ is responsible for the clinical differences in pain and disability observed between the two groups. Mientjes and Frank^3^ assessed a small sample (n=8 per group) and although reported significant differences between CLBP and asymptomatic groups during challenged standing conditions, these differences were small (less than 2 mm) and similar to those of both the current study and Brumagne *et al*’s study.^1^ Furthermore, Mientjes and Frank^3^ report a mean pain score of 0.5 in the ‘asymptomatic’ group raising concerns that the asymptomatic data may not be a true representation of a pain-free population.

The CoP parameters assessed in a research study may influence the reliability of results. CoP velocity consistently demonstrates the best overall reproducibility of all CoP parameters in the short and long terms,^12 30^ hence, findings from this parameter are likely to provide more reliable conclusions to those gained from CoP_RMSEAP_ data or other CoP parameters. The current study demonstrated similar CoP_VELAP_ in people with and without CLBP, whereas previous research has demonstrated reduced^4 31^ (n=24 and 22 per group, respectively) and increased^2 32 33^ (n=12, 12 and 10 per group, respectively) CoP velocities. These mixed results suggest it is likely that research demonstrating no difference between groups has been conducted, however, due to publication bias may not have gained acceptance for publication. Interestingly, the studies conducted with the greater sample size demonstrate poorer postural control in the asymptomatic groups, not the CLBP groups. Furthermore, findings from previous research^31 34^ highlight that the small differences observed between groups in this study may be due to random error associated with the reliability of the measurement technique and not clinical change.

Differences in participant demographics (eg, age,^2 31^ gender^33^ or disability^4^) and methodological design (eg, trial duration and repetitions^4 30 32^) make it difficult to directly compare study findings. Due to the numerous factors which may contribute to the variation in CoP outcomes reported, comparison of one study data with another is likely to reveal potential differences; however, choice of outcome measures and the number and duration of trials conducted in the current investigation improves the likelihood that data collected are reliable.

### Gait

No differences were detected in spatiotemporal parameters between groups. In support of the current study findings, Al-Obaidi *et al* 5 and Simmonds and Claveau^35^ demonstrated no difference in cadence and self-selected walking speed, respectively, between people with and without CLBP (with a similar age and gender to those in the current study). However, research investigating participants with similar self-reported pain (mild to moderate) to the current study demonstrated reduced walking speed,^5–8^ stride time^9 10^ and stride length^5 6^ in people with LBP. The current study averaged data from three trials for each participant, aiming to improve reliability,^12^ whereas other studies analysed data from only one walking trial,^5 8 9^ possibly reducing data reliability. In addition, where other studies investigated predominantly^6^ or all male participants,^8 9^ the current study assessed male and female participants, enabling findings to be more representative of a general population. Furthermore, the current study assessed participants walking on normal ground, as opposed to on a treadmill,^7 9 10^ hence, the current study findings are likely to be more representative of a natural walking pattern. These factors increase confidence that the current results are a more reliable and valid representation of gait in CLBP than that reported in previous research.^5–10^

In contrast to the current study, previous research has reported reduced hip range of movement in people with LBP during gait compared with asymptomatic individuals.^9^ This may be due to co-contraction of muscles crossing the hip and pelvic region^36^ limiting hip movement, or from participants reducing step length, and hence hip range, in an attempt to reduce potentially detrimental ground reaction forces at heel strike.^15 37^ Reduced hip range demonstrated by Vogt *et al* 9 occurred during treadmill gait, hence may not be representative of natural gait.^38^ Furthermore, Vogt *et al* 9 assessed hip range by attaching an electrical goniometer to the greater trochanter. This method of assessment provides less reliable data than the retroreflective marker system used in the current study^39 40^; again increasing confidence that the current results are likely a more valid representation of gait in people with CLBP. In the current study, due to the lack of difference in stride length between CLBP and asymptomatic individuals, the similar range of hip movement between the two groups was an expected finding.

### Strengths and limitations

The authors did not conduct a formal sample size calculation using MCID data due to the absence of reported MCID data within the literature. However, SEs of measurements from repeatability studies for similar sample populations are reported in the literature for the more reliable postural stability outcome measure of CoP_VELAP_.^41^ If minimal detectable change (MDC) is substituted for MCID in a sample size calculation (where alpha=0.05, beta=0.8, MDC for CoP_VELAP_=5.4 mm, SD of groups=1.09 and 1.52 where groups contain equal number of participants) this suggests that six participants would need to be recruited.

The authors note the convenience sample recruited in this study for the asymptomatic participants may not be representative of the general population; however, potential asymptomatic participants were required to meet inclusion and exclusion criteria with a view to reducing this potential source of sampling bias. Sampling bias may have been reduced in the symptomatic sample as recruitment of participants occurred more broadly from a population with CLBP in multiple recruitment sites.

Although participants were matched for age and gender, the authors note that unaccounted confounders, such as anthropometric factors, level of physical activity, or kinesiophobia, may have influenced study results. Given the small sample size in the current study, multivariate modelling was deemed inappropriate. Research investigating the influence of anthropometric factors (including body height, limb and trunk length, and body mass) on postural balance concluded postural balance assessed with eyes open and closed is only slightly^42^ and moderately^43^ influenced by these anthropometric variables; the variables that most influenced postural balance being height and body mass index. The similarity of height and weight between groups in the current study (table 1) is therefore reassuring.

The current study recruited CLBP participants from clinical populations,^21^ who had sought medical opinion regarding their symptoms, hence, represented a typical population treated within physiotherapy departments. Previous research has recruited participants from alternative sources such as university populations,^4^ which may not be representative of the subgroup of CLBP individuals who seek medical guidance; hence caution should be taken if relating findings from such studies to a person with CLBP who is attending for treatment.

### Further research

The velocity of the CoP is reported as the most reliable CoP parameter; however, it is unclear if this measure is the most appropriate to detect difference in postural stability. Hence, a difference in postural control between the symptomatic and asymptomatic groups may have been present, but not detected. Alternative balance measures could be investigated, such as the forward reach test to determine whether more functional or challenging outcomes possess the necessary discriminatory value to detect differences in balance in people with and without CLBP and assist in confirming whether such differences exist.

### Clinical implications

Based on the findings of this study, clinicians can be informed that standing postural stability, kinetic, kinematic and spatiotemporal parameters of gait in people with and without mild to moderate CLBP may not differ, and that treatments directed at influencing postural stability (eg, standing on a wobble board) or specific parameters of gait may be an unnecessary addition to a treatment programme.

## Conclusions

In contrast to previous research, this study suggests that people with mild to moderate CLBP may present with similar standing postural control, hip moments and range of movement, and spatiotemporal parameters of gait to asymptomatic individuals.
