# Supplementary material for: Comparison of standing postural control and gait parameters in people with and without chronic low back pain: a cross-sectional case–control study
Source: BMJ Open Sport Exerc Med. 2018 Jan 23;4(1):e000286. doi: 10.1136/bmjsem-2017-000286 (PMC5783032; doi:10.1136/bmjsem-2017-000286)
Supplement: Supplementary file 1 [file bmjsem-2017-000286supp001.pdf]

Appendix 1

Centre of pressure calculations

Centre of pressure (CoP) calculations were made from the output from two force plates inset in the laboratory floor. The figure below demonstrates the x, y and z axes of the force plates. Yellow arrow represents the x-axis; green arrow, the y-axis; and orange arrow, the z-axis.

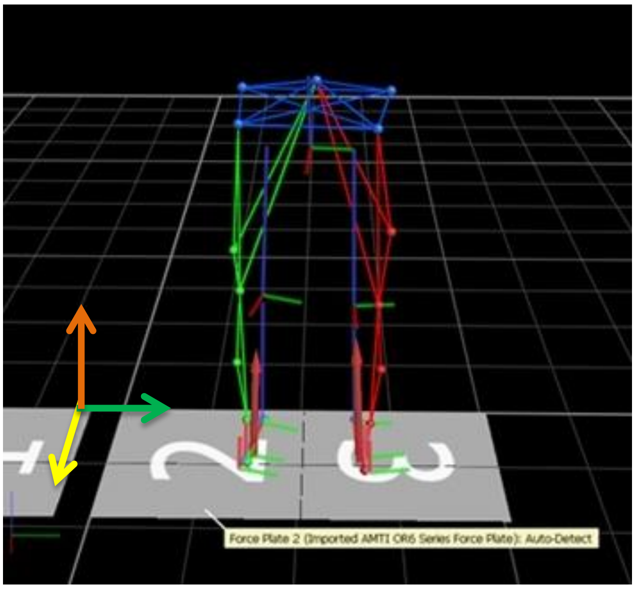

The x-coordinate of the CoP was calculated under each limb from the moments and forces produced by each plate with respect to the origin of the laboratory space, as follows:

$$x_{CoPl\_i} = \frac{-M_{yl\_i}}{F_{zl\_i}} + plate\ origin_{xl}$$

$$x_{CoPr\_i} = \frac{-M_{yr\_i}}{F_{zr\_i}} + plate\ origin_{xr}$$

where x_{CoPl_i}, x_{CoPr_i} are x- coordinates of the CoP under the left and right feet at time point i, and M_{yl_i}, M_{yr_i}, F_{zl_i}, F_{zr_i} are directional components of the moments and forces acting on the body from each force plate. These coordinates are expressed relative to the global coordinates of the laboratory space by a translation between the origin of the force plate and the origin of the laboratory ($plate\ origin_{xl}$, $plate\ origin_{xr}$) .

The x-coordinate of the CoP of the whole body was calculated by multiplying the x-coordinate of the CoP for each limb by the fraction of the total vertical force (F_z) acting through that limb, and adding the two terms together, as follows:

$$x_{CoP\_i} = x_{CoPl\_i} * \left(\frac{F_{zl\_i}}{F_{zl\_i} + F_{zr\_i}}\right) + x_{CoPr\_i} * \left(\frac{F_{zr\_i}}{F_{zl\_i} + F_{zr\_i}}\right)$$

where x_{CoP_i} is the x-coordinate of the CoP of the whole body.

Calculation of the root mean squared error of the centre of pressure in the antero-posterior direction ($CoP_{RMSE\ AP}$)

The root mean squared error of the CoP in the antero-posterior direction (x-direction) is given by:

$$CoP_{RMSE\_AP} = \sqrt{\sum_{i}^{N} \frac{\left(x_{CoP\_i} - \overline{x_{CoP\_i}}\right)^2}{N}}$$

where $\overline{x_{CoP\_i}}$ is the mean position of the x-coordinate of the CoP, and N is the number of time points in the trial.

Calculation of centre of pressure velocity in the antero-posterior direction ($CoP_{VEL\ AP}$)

The mean velocity of the CoP in the antero-posterior direction (x-direction) is given by:

$$CoP_{VEL\_AP} = \sum_{i} \frac{\left|x_{CoP\_i} - x_{CoP\_i-1}\right|}{N} * f_s$$

where f_s is the data sampling frequency.
